# A Novel Device for Grasping Assessment during Functional Tasks: Preliminary Results

**DOI:** 10.3389/fbioe.2016.00016

**Published:** 2016-02-22

**Authors:** Ana Carolinne Portela Rocha, Eloisa Tudella, Leonardo M. Pedro, Viviane Cristina Roma Appel, Louise Gracelli Pereira da Silva, Glauco Augusto de Paula Caurin

**Affiliations:** ^1^Research in Motion Analysis Laboratory, Department of Physical Therapy, Federal University of São Carlos, São Carlos, Brazil; ^2^Mechatronics Group, Department of Mechanical Engineering, Federal University of São Carlos, São Carlos, Brazil; ^3^Mechatronics Group, Department of Mechanical Engineering, São Carlos School of Engineering, University of São Paulo, São Carlos, Brazil

**Keywords:** grasp quality evaluation, upper limb assessment, children rehabilitation, image processing, neurologic rehabilitation

## Abstract

This paper presents a methodology and first results obtained in a study with a novel device that allows the analysis of grasping quality. Such a device is able to acquire motion information of upper limbs allowing kinetic of manipulation analysis as well. A pilot experiment was carried out with six groups of typically developing children aged between 5 and 10 years, with seven to eight children in each one. The device, designed to emulate a glass, has an optical system composed by one digital camera and a special convex mirror that together allow image acquisition of grasping hand posture when it is grasped and manipulated. It also carries an Inertial Measurement Unit that captures motion data as acceleration, orientation, and angular velocities. The novel instrumented object is used in our approach to evaluate functional tasks performance in quantitative terms. During tests, each child was invited to grasp the cylindrical part of the device that was placed on the top of a table, simulating the task of drinking a glass of water. In the sequence, the child was oriented to transport the device back to the starting position and release it. The task was repeated three times for each child. A grasping hand posture evaluation is presented as an example to evaluate grasping quality. Additionally, motion patterns obtained with the trials performed with the different groups are presented and discussed. This device is attractive due to its portable characteristics, the small size, and its ability to evaluate grasping form. The results may be also useful to analyze the evolution of the rehabilitation process through reach-to-grasping movement and the grasping images analysis.

## Introduction

Reaching and grasping objects is the base for acquiring more complex manual abilities, which involves a combination of reach, grasp, transport, and release (Coluccini et al., 2007). Manual interaction with objects begins in an early stage of human motor development. Around the age of 4 months, infants are able to direct the arm toward a target of interest and grasp it (von Hofsten and Lindhagen, 1979). Nonetheless, in this period, upper limb movements that result in grasping are marked by a high variation in velocity, in amplitude and in duration along the corresponding path (von Hofsten, 1991). Kinematic analysis of immature reaching in infants reveals an irregular and awkward trajectory, with a great number of accelerations, decelerations, and corrections, known as movement units (von Hofsten and Lindhagen, 1979; Shumway-Cook et al., 2003). In the following months, movements become progressively smoother and fluent by showing a straighter trajectory, decreasing the number of movement units and increasing execution velocity (von Hofsten, 1991; Thelen et al., 1993). Until the end of the 10th year, the development of prehensile abilities is completed in typically developing children, which is demonstrated by well-established kinematic patterns (Kuhtz-Buschbeck et al., 1998; Paré and Dugas, 1999).

Daily and occupational activities require a wide spectrum of manual skills, such as self-care, feeding, and scholarly activities for children, for example writing, painting, cutting, etc. Especially in childhood, perceptual-motor development is intimately related to the cognitive development, since exploratory actions on objects is essential to construct knowledge (Rosengren et al., 2003; Corbetta and Snapp-Childs, 2009; von Hofsten, 2009). For this reason, motor, sensorial, and/or mental disabilities caused by various risk factors may lead to delay in both physical and intellectual development. Some of these factors include prematurity and diagnosed conditions, such as congenital or acquired encephalic injuries, like cerebral palsy; cerebral vascular accident and brachial plexus palsy; genetic syndromes, like Down syndrome; and congenital malformations, like arthrogryposis multiplex congenital (King et al., 1992). Part of these causes demand long-term processes of rehabilitation addressed to the use of the hands (Shumway-Cook et al., 2003; Coluccini et al., 2007; Jaspers et al., 2009).

Evaluation is a decisive step in programs to improve or recover functional performance. Regarding upper limb functional assessment, diverse validated tools are used for measuring rehabilitation effects as soon as motor performance is directly observed through movements and tasks requested for the patient (DeMatteo et al., 1993; Randall et al., 2001; Wolf et al., 2001; Henderson et al., 2007; Uswatte et al., 2012a,b; Rosa-Rizzotto et al., 2014; Santos et al., 2015). There are other devices such as dynamometers, used for taking isolated measurements of grasp strength, and gonio­meters, used for measuring joint angles. None of these integrates quantitative and qualitative variables or enables simultaneous acquisition of data during the evaluation with functional movement procedures.

Clinicians and researchers have been concerned in a more objective and less examiner-dependent standardization of protocols and assessment instruments, which warrant suitable reproducibility among measurements (Coluccini et al., 2007; Jaspers et al., 2009, 2011). In this sense, tridimensional motion analysis is considered a more sensitive tool to evaluate interventions efficacy, the gold standard within quantitative assessment methods (Chang et al., 2005; Jaspers et al., 2009).

Despite having countless advantages, kinematic methodologies usually require wide infrastructure and, from the economic point of view, sophisticated and expensive equipments. Consequently, the development of portable and non-invasive devices that enable detailed analyses of the motion in any environment, either in therapeutic, domiciliary or educational context, is extremely important.

This research introduces the Grasp and Upper Limb Motion Sensor (GULM Sensor), a device recently developed by the Mechatronics Laboratory at the São Carlos School of Engineering at the University of São Paulo (USP), in partnership with the Research in Movement Analysis Laboratory at the Federal University of São Carlos. This device is attractive due to its portability, small system size, and capacity to assess qualitatively and quantitatively the upper limb movements and grasping postures during interaction tasks. Its purpose is to assist the measurement of outcomes in hand and upper limb rehabilitation process.

In addition, movement analysis researches demand standardization of the task or activity which well represents subjects’ functional capabilities. Simulating “drinking water” with a glass enables a standardized assessment before and after intervention. In related works, consistent kinematic patterns were found in typically developing children, with significant differences between these and children with cerebral palsy. It is possible to divide the reach and grasp cycle of this task in phases, such as reaching and grasping a cup on a table, transporting it to the mouth and returning it to the starting position (Butler et al., 2010a). A series of upper limb movements, which involve its main joints are generated from this and thereby present the potential of identifying and differentiating types of motor disabilities (Butler et al., 2010b).

Thus, once the movement protocol has been chosen, experiments with the device must be conducted primarily with typically developing children in order to establish the reliability of the procedures (Jaspers et al., 2011) and to build control data to further analysis with of experiment with atypical children. Summarily, the aim of this paper is to present this novel device and its potential analysis through experiments and their preliminary results.

## Materials and Methods

### The Grasp and Upper Limb Motion Sensor

**Figure 1:**
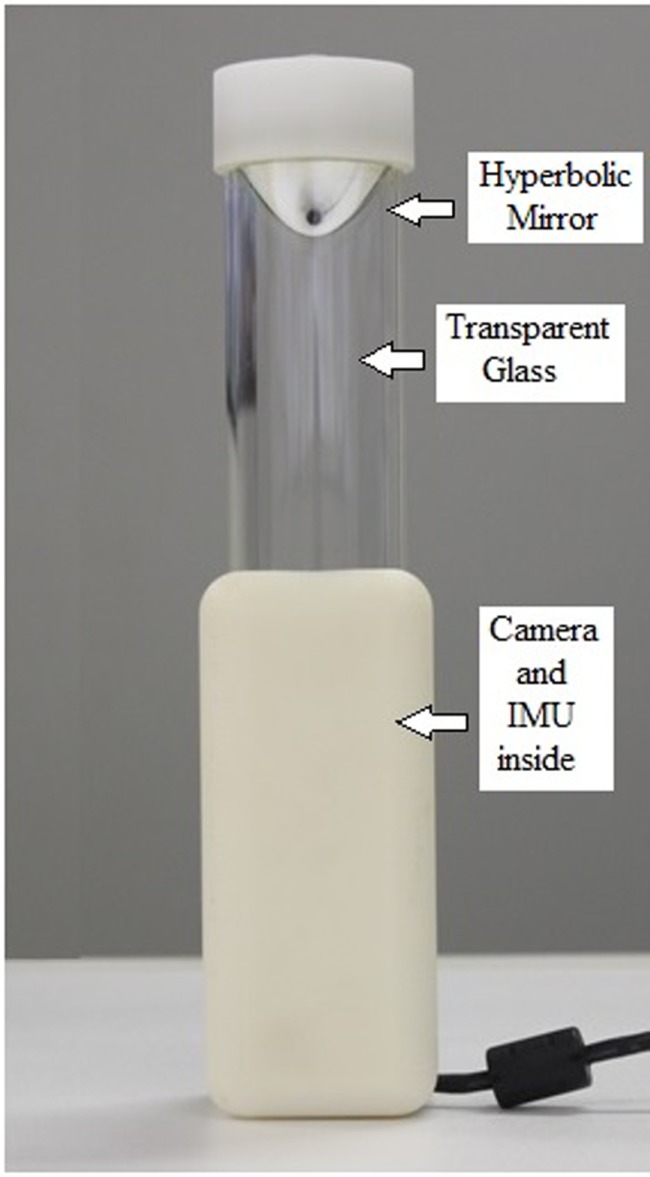
**GULM Sensor overview: optical system and Inertial Measurement Unit (IMU)**.

For this study, a prototype of the GULM Sensor, which resembles a transparent glass (150 mm × 50 mm × 3 mm, 550 g) was designed and tested. The device is presented in Figure 1. The GULM Sensor is based on the combination of two sensor-subsets: a vision system and a motion sensor. The upper part of the device has a hyperbolic mirror mounted just below the cap, which is fastened on a transparent hollow glass cylinder. The prismatic base houses a camera, optical lenses, and an Inertial Measurement Unit (IMU).

### Vision System Hardware

**Figure 2:**
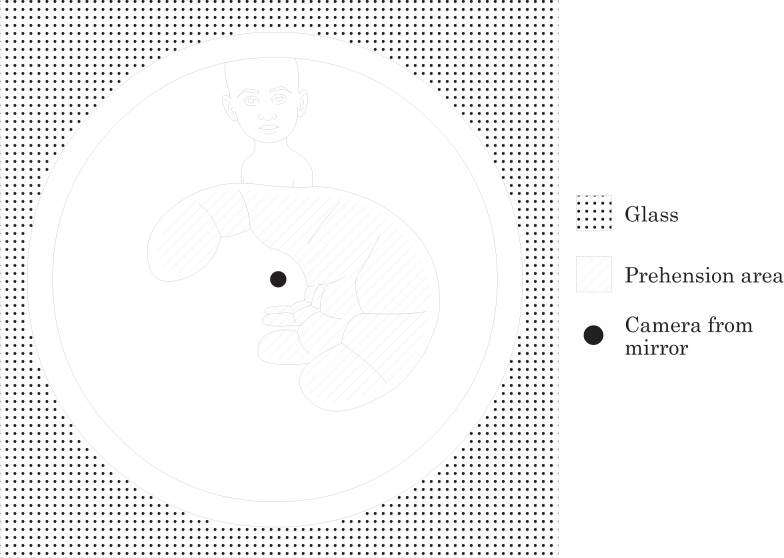
**Hand image of the transparent cylinder reflected on the hyperbolic mirror and captured by the camera**.

This system allows the assessment of the grasping hand posture. Its design was inspired on omnidirectional sensors (Grassi Junior and Okamoto Junior, 2006) that are commonly used in mobile robotics. It uses a hyperbolic aluminum mirror machined in a high precision lathe. The mirror projects a 360° cylindrical grasping image to a digital camera (BASLER, acA1300-30gm) with 1280 × 960 pixels of resolution and acquisition rate of 100/60 frames per second. Data are transferred using a dedicated USB connection directly to a host PC. Access to the image data is provided by an image acquisition software (Point Grey, FlyCap Viewer 2.6). Figure 2 sketches a typical hand surface image when the transparent cylinder is grasped, and the hand reflected on the hyperbolic mirror is captured by the camera. The procedure to extract the posture of the hand when grasping the cylinder glass, based on the algorithm proposed by Grassi and Okamoto (Grassi Junior and Okamoto Junior, 2006), is described in Section “Image Processing Method.”

### Inertial Measurement Unit

A Motion Sensor (LP-RESEARCH LPMS-B, Bluetooth 2.1 + EDR, 2.412–2.484 GHz), was chosen to integrate the GULM. It is a miniature, multi-purpose IMU. The unit can measure the orientation in 360° in all three global axes.

For measuring the orientation of the device, the sensor uses three internally located units: a 3-axis gyroscope, which detects angular velocity; a 3-axis accelerometer, which detects linear acceleration in *x*, *y* and *z*; and a 3-axis magnetometer, for measuring direction of the earth magnetic field.

The IMU communicates with an external computer transmitting data through Bluetooth (wireless) communication. LPMS firmware makes a pre-processing of the sensor signals and displays the results in a standard *text file*.

### Ethical Procedures and Participants

Experiments were conducted with the approval of the Human Research Ethics Committee from Federal University of São Carlos (protocol number 508 804). All the guardians signed the Legal Consent Form and the children signed the Consent Assent. This study follows the Helsinki protocol.

A total of 47 healthy, full-term born, and typically developing children (22 boys and 25 girls, aged 5–10 years, mean age 7.49 ± 1.73 years, all with high hand dominance) participated in this study. They were randomly selected from schools of a medium city in the state of São Paulo, Brazil. Children and their respective guardians did not declare any history of psychomotor developmental delay or orthopedic surgeries for the upper limbs; musculoskeletal or neurological conditions affecting upper limbs function; cognitive impairment that would prevent understanding of the requested task; or disability in the visual and/or auditive systems. For the analyses, they were divided in six groups according to their ages, with seven to eight children in each one.

**Figure 3:**
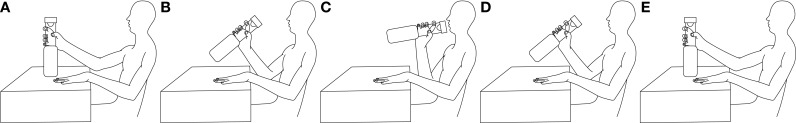
**Experiment setup: while remaining seated in front of a table, (A) the child reaches forward with his/her right hand until he/she grasps the glass, (B) lifts it off the table, and (C) transports it to the mouth**. After that, **(D)** he/she returns the device and **(E)** releases it on the table.

During the test procedure, children remained seated in an adjustable chair in front of a table with their arms and palms resting on its top, hips, and knees were flexed in 90° and both feet flat on the ground. Upper limbs were positioned in a neutral rotation of shoulders, forearms pronated, and the wrists held as well in a natural position (Butler et al., 2010a). Each child was instructed to simulate the task of drinking a glass of water, as shown in Figure 3. The device was placed on the table in front of the participant. From the initial position, the child reaches forward to grasp the device and transports it up to his/her mouth (until it touches his/her lower lip), and then returns it to the starting position releasing the device (Butler et al., 2010a,b). Three trials were carried out with an interval of 5 s between two consecutives.

### Spatio-Temporal Variables

Commonly, patterns and movement efficiency of the upper limb in typical and atypical children are described in terms of spatio-temporal and angular variables (von Hofsten, 1991; Chang et al., 2005; Carvalho et al., 2007; Coluccini et al., 2007; Butler et al., 2010a,b; Jaspers et al., 2011; Butler and Rose, 2012). Those variables can be obtained from the IMU, such as the duration of each phase and of the total task, velocity parameters (average velocity, velocity peak), number of movement units, straightness index, and angular variation of the device in *x*, *y*, and *z* axes.

The number of movement units is calculated by the analyses of the velocity profile and is defined as the difference between a maximum velocity and a minimum that is greater than a predetermined threshold (von Hofsten, 1991; Chang et al., 2005). In a previous study, with the same task a threshold of 40 mm/s was used to obtain each movement unit (Butler et al., 2010b).

Straightness index (Rowlands, 2007; Choi et al., 2010) is the ratio between the lowest distance which the device can be moved in the sagittal plane (distance in a straight line between the initial position of the device on the table and the final position close the mouth) and the real traveled distance. It demonstrates how straight is the path of the movement. As the index is closer to one, the path is straighter.

Energy expenditure is a common outcome for the estimating of physical activity level in children, young, and adults, easily provided by accelerometers (Rowlands, 2007; Choi et al., 2010), such as those presented in the IMU.

Data for each trial (a complete cycle of drinking simulation) were extracted from a standard *txt* format file, imported into Excel spreadsheets and used to calculate the variables in Excel or Matlab.

### Image Processing Method

Image processing is responsible for segmentation, the separation of information that is related to grasping and the preparation of the image for extracting geometrical information such as grasping area. The challenge in this phase is inherent due to the attempt of overcoming the issues we face in a computer vision system, such as variations on lighting conditions and sheen, clothing, not relevant parts of the body appearing in the scene, quality of lenses and cameras, equipment calibration, among other features.

This study has considered some of the main segmentation techniques, as described by Erol et al. (2007). Some segmentation techniques were tested, including Thresholding, Simple Subtraction, Background Subtraction, Edge Detection, and K-Means Clustering (Gonzalez and Woods, 2010).

The K-Means Clustering segmentation based on skin color analysis was adopted because of its best consistency in color variation detection.

The segmentation algorithm first converts the RGB color image into an image in the L*a*b* color space also known as CIELAB. Then, the algorithm classifies the image colors in L*a*b* using K-means cluster analysis, considering three clusters and Euclidean distance.

Clustering is a way of separating groups of objects, which is done by identifying collections of objects in the image that are similar to each other and separating the different objects belonging to other clusters. It finds partitions such that objects within each cluster are as close as possible to each other and as far as possible from objects in other clusters.

**Figure 4:**
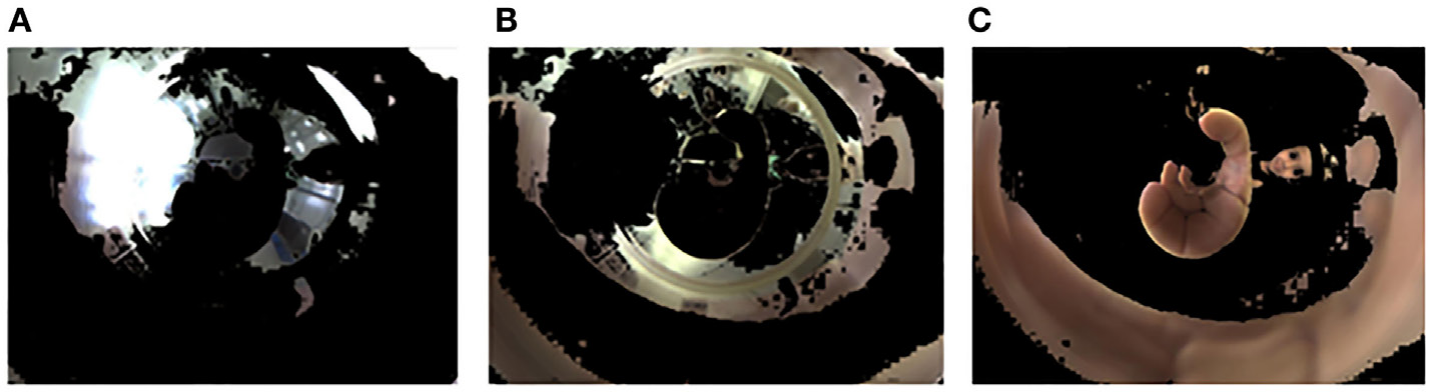
**(A)** Objects in cluster 1; **(B)** Objects in cluster 2; **(C)** Objects in cluster 3.

Subsequently, for every input object, the algorithm returns an index corresponding to a cluster. Then using the index, the algorithm separates objects by their colors illustrated in Figure 4.

**Figure 5:**
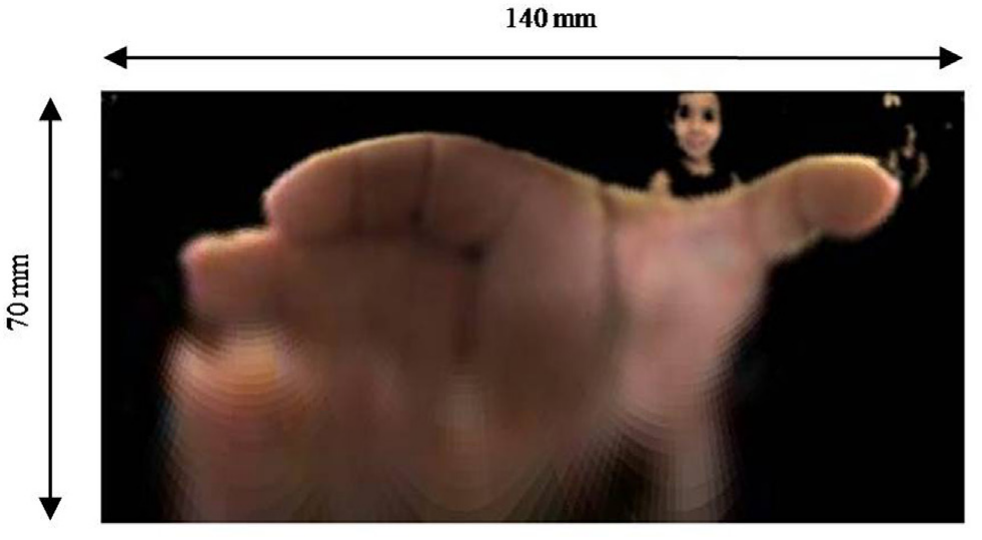
**Panoramic view of Figure 4C**.

After this clustering process, the image selected is the one that clusters pixels with the skin color (Figure 4C). To conclude the method, the mirrored image is transformed into a panoramic one applying the algorithm described by Grassi Junior and Okamoto Junior (2006). The picture presented in Figure 5 shows the panoramic view of Figure 4C. The picture represents the cylinder surface which area is 140 mm × 70 mm. The total length of the cylinder is 100 mm, however, 30 mm from its bottom were rejected due to loss of information. A lot of visual information concentrated close to the center of the mirror is represented by a few pixels of the image, resulting in a final image with poor resolution close to its bottom.

The image processing and the generation of the panoramic view algorithms were implemented in Matlab.

### Statistical Analysis

SPSS Statistics Software, version 17.0 (IBM^®^, Chicago, IL, USA) was used for statistical analysis. Descriptive analyses and tests to verify data normality (Shapiro–Wilk and Kolmogorov–Smirnov) preceded the comparative analyses. Therefore, parametric or non-parametric tests were used depending on the nature of the variable. Comparisons between gender and the phases of transport and return were performed using one of these tests: independent-samples *t*-test or Mann–Whitney test. ANOVA one-way test with Tukey *post hoc* test or the Kruskal–Wallis test with Mann–Whitney *post hoc* test and Bonferroni adjustment for multiple comparisons were used to compare variables by age. A significance level of *p* < 0.05 was adopted.

## Results

### Kinematic and Kinetic Analysis

**Figure 6:**
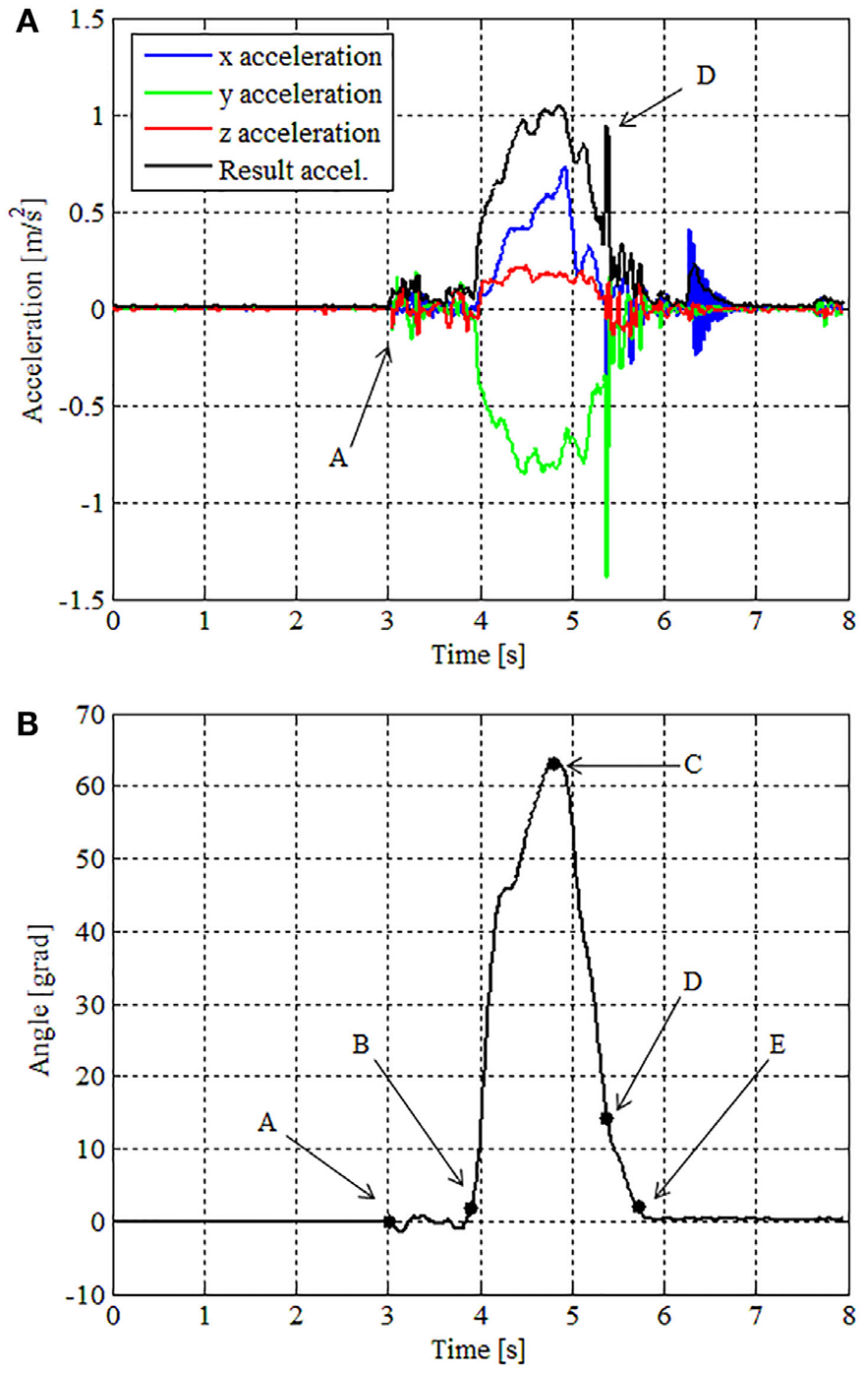
**(A)** Accelerations as a function of time for the trial taken as example; **(B)** Euler angle Y (sagittal plane) for the same trial of **(A)**.

The curves shown in Figure 6A present an example of accelerations along time for one trial repetition chosen randomly. Point A indicates the acceleration peak when the hand touches the object for the first time. Numerically the acceleration peak is detected when the resultant of the three acceleration components is greater than an *ad hoc* threshold of 0.02 m/s^2^. This resultant acceleration is calculated taking into account and compensating gravitational acceleration. Before the calculation, the acceleration is submitted to a low-pass filter of 15 Hz.

After the contact instant, the movement beginning can be identified analyzing Euler angle Y provided by the IMU. These angles represent the angular displacement of the object on the sagittal plane. Point B indicates this moment in Figure 6B. In this example, the transport movement starts at 0.170 ms after the first contact with the glass. In this paper we call this period as “accommodation phase,” i.e., the time required to accommodate the hand in a stable pretension state.

Following the task evolution, after the movement beginning is identified, the next instant of interest is the time when the glass approaches the mouth. This moment is defined as the state when the motion amplitude reaches its maximum, which is identified with the character C in Figure 6B. We suggest the correlation of this moment with the inversion of the movement, which corresponds to the end of the transport phase and beginning of the return phase, i.e., the third phase.

Analyzing data from all the subjects, we observed that the end of the return phase may be observed by the occurrence of two different events:

(1) Return to the initial orientation (initial Euler angle Y) – this is detected when the Euler angle is smaller than an empiric threshold previously used to define the movement initiation. This is represented by character E in Figure 6B;
(2) Contact with the table surface – this is detected analyzing the second acceleration peak illustrated in Figure 6 with the character D.

We evaluated these two events using a two-way ANOVA in order to find the correlation among the events with age or gender, but the results were inconclusive. For evaluation purposes we consider the contact with the table as the end of the return phase. We assume, as a hypothesis, that the contact helps the volunteer to stabilize the object and as a consequence stability and control analysis are no longer effective when the object in contact with the table.

As a summary of the kinematic analysis, the instants identified were: A – contact establishment; B – movement beginning; C – movement maximum amplitude; D – movement end. These instants divide the three phases of the task: Phase I – accommodation of the hand for a stable grasp; Phase II – transport of the glass from the table to the mouth; and Phase III – return of the glass from the mouth back to the table. Analyses were performed comparing the execution time of the phases relative to the total time of the task for each experiment participant.

**Figure 7:**
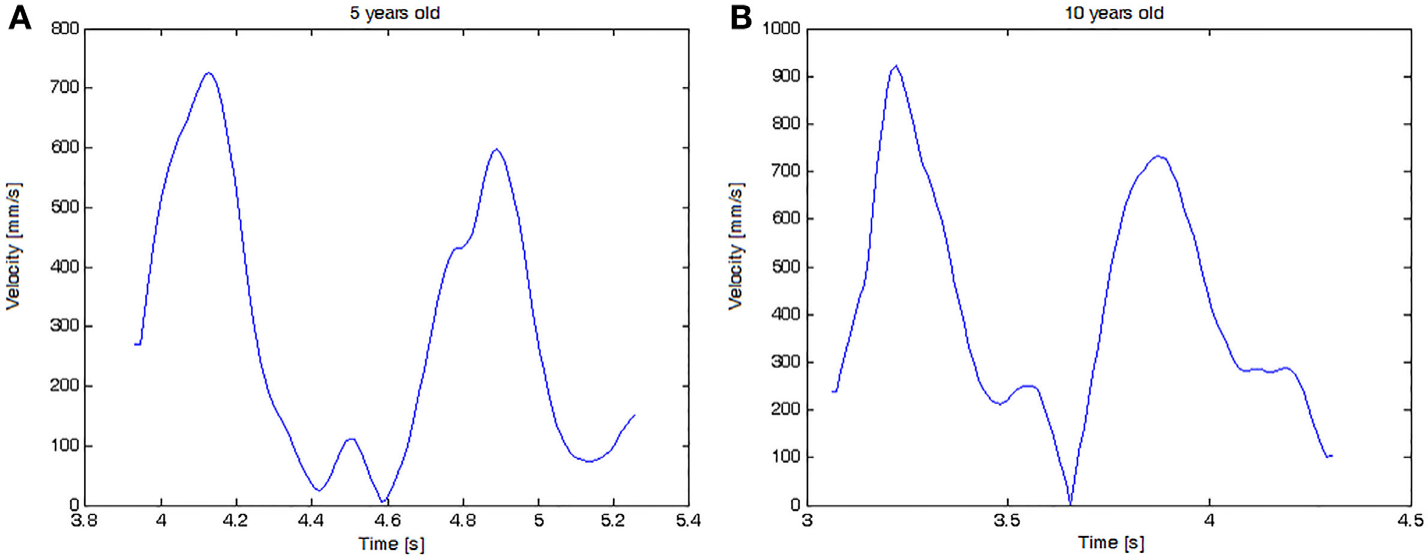
**Velocity graph of the complete task cycle for two children: (A) a 5-year-old child with 3 movement units and (B) a 10-year-old child with 2 movement units**.

Additionally, considering the movement unit metric (well established in the field of motion analysis) Figure 7 illustrates an example of movement units that can be obtained from velocity graphs of a complete cycle. It shows the evaluation of two children, a 5-year-old boy (with three movement units) and a 10-year-old boy (with 2 movement units).

### Kinematic Analysis

Considering all the subjects, the average total duration of the cycle was 2.55 ± 0.6 s. Accommodation phase was 13.33% (±6.05), transport phase was 43.24% (±7.41), and return phase 43.43% (±7.73).

In detail, boys completed the task faster than girls (*p* = 0.013), however the proportion of each phase was similar for both genders (Table 1). Considering energy expenditure, no significant differences were found between genders for both transport (*p* = 0.480) and return (*p* = 0.313). Comparing phases in each gender, girls demonstrating a higher energy expenditure in the return phase (*p* = 0.007).

**Table 1:** **Spatial-temporal parameters for boys and girls**.

	Boys (*n* = 22)	Girls (*n* = 25)	*p*-value
Total duration (s)	2.41 ± 0.53	2.66 ± 0.64	0.013*
Accommodation phase (%)	13.67 ± 6.10	13.03 ± 6.03	0.5221
Transport phase (%)	43.17 ± 6.62	43.29 ± 8.09	0.9271
Return phase (%)	43.15 ± 6.88	43.68 ± 8.45	0.687^1^
Energy expenditure**(×10^3^)			
Transport	97.02 ± 115.88	77.78 ± 66.68	0.480^1^
Return	156.60 ± 274.78	108.69 ± 85.78	0.313^1^
*p-*value	0.573^1^	0.007^1^	
**Straightness index**			
Transport	0.93 ± 0.02	0.94 ± 0.03	0.192^1^
Return	0.92 ± 0.08	0.92 ± 0.05	0.852^1^
*p-*value	0.310^1^	0.083^1^	

*n*, number of participants in each group. Values are expressed as mean ± SD. *Independent-samples *t* test; 1Mann–Whitney test. Significance level corresponds to *p* < 0.05. **Estimation based on the square of the angular velocity.

In Table 2, it can be noticed for accommodation phase a trend of time decreasing as age increases, but without any statistical significance (*p* = 0.450). Five years old children were faster than 8 years old (*p* = 0.002) and 10 years old (0.003). Furthermore, in the transport phase, 5-year-old children presented higher energy expenditure than all the another ages. The same phenomenon was observed regarding ages of 8–10 years for the return phase. Those were 6–7 years either presented higher energetic spending than those of 9 and 10 years.

**Table 2:** **Spatial-temporal parameters regarding age of the participants**.

	Age (years)						*P-*value
	5 (*n* = 8)	6 (*n* = 8)	7 (*n* = 8)	8 (*n* = 7)	9 (*n* = 8)	10 (*n* = 8)	
Total duration (s)	2.30 ± 0.82^a,b^	2.66 ± 0.63	2.43 ± 0.58	2.69 ± 0.37^a^	2.67 ± 0.68	2.54 ± 0.29^b^	0.022^1^
Accommodation (%)	16.21 ± 8.87	13.77 ± 6.17	14.25 ± 6.56	11.76 ± 3.28	12.01 ± 4.41	11.80 ± 4.25	0.450^1^
Transport (%)	40.94 ± 8.92	43.31 ± 6.41	44.16 ± 4.94	41.34 ± 8.45	44.86 ± 7.54	44.56 ± 7.50	0.311*
Return (%)	42.85 ± 10.24	42.91 ± 7.14	41.59 ± 5.87	46.90 ± 7.93	43.13 ± 7.07	43.64 ± 7.30	0.318*
Energy expenditure**(×l0^4^)							
Transport	20.13 ± 15.98^c,d,e,f,g^	8.46 ± 5.11^c,h^	64.89 ± 4.10^d^	6.22 ± 6.93^e^	6.41 ± 4.16^f^	4.49 ± 3.22^g,h^	<0.001^1^
Return	24.30 ± 26.16^i,j,k^	15.01 ± 11.45^l,m^	13.00 ± 9.66^n,o^	16.22 ± 36.09^i^	5.94 ± 3.69^j,l,n^	4.51 ± 2.04^k,m,o^	< 0.001^1^
**Straightness index**							
Transport	0.94 ± 0.04	0.93 ± 0.02	0.94 ± 0.02	0.93 ± 0.02	0.94 ± 0.02	0.94 ± 0.02	0.562^1^
Return	0.92 ± 0.08	0.92 ± 0.03	0.93 ± 0.03	0.89 ± 0.13	0.92 ± 0.03	0.94 ± 0.02	0.015^1^

Values are expressed as mean + SD. 1Kruskal–Wallis test with Mann–Whitney *post hoc* test and Bonferroni adjustment. *ANOVA one-way test with Tukey *post hoc* test. Significance level corresponds to *p* < 0.05. Significant difference: ^a^*p* = 0.002; ^b^*p* = 0.003; ^c^*p* < 0.001; ^d^*p* < 0.001; ^e^*p* < 0.001; ^f^*p* < 0.001; ^g^*p* < 0.001; ^h^*p* = 0.002; ^i^*p* = 0.001; ^j^*p* < 0.001; ^k^*p* < 0.001; ^l^*p* = 0.001; ^m^*p* < 0.001; ^n^*p* = 0.001; ^o^*p* < 0.001. **Estimation based on the square of the angular velocity.

Comparing genders at the age of 9, return phase was longer for boys (*p* = 0.046). On the other hand, at 10 years old it was longer for girls (*p* = 0.008). In transport phase, boys of 5 years had higher energy expenditure than their pairs of other ages, while girls of 5 years had higher than those of 8 and 10 years. Girls had higher straightness index than boys at the age of 10, in both, transport and return phases (*p* < 0.001). Surprisingly, boys of 5 years had the greatest value of straightness index, but only different of those with 8 (*p* = 0.003) and 10 years (*p* = 0.001). Otherwise, girls of 5 years had the least value with no significant difference in relation to the other ages. Girls at 10 years had higher straightness index than those at 8 (*p* = 0.002).

### Preliminary Grasping Posture Analysis

Together with the IMU data, images from the mirror are also acquired during the task execution with a frame per second rate of 100/60 (100/60 Hz). The images contain the mirrored posture of the hand grasping the cylinder. Applying the image processing method presented in Section “Image Processing Method” and the panoramic view transformation proposed by Grassi and Okamoto (Grassi Junior and Okamoto Junior, 2006), it is possible to assess the hand posture in a metric panoramic image in every 1.67 s.

**Figure 8:**
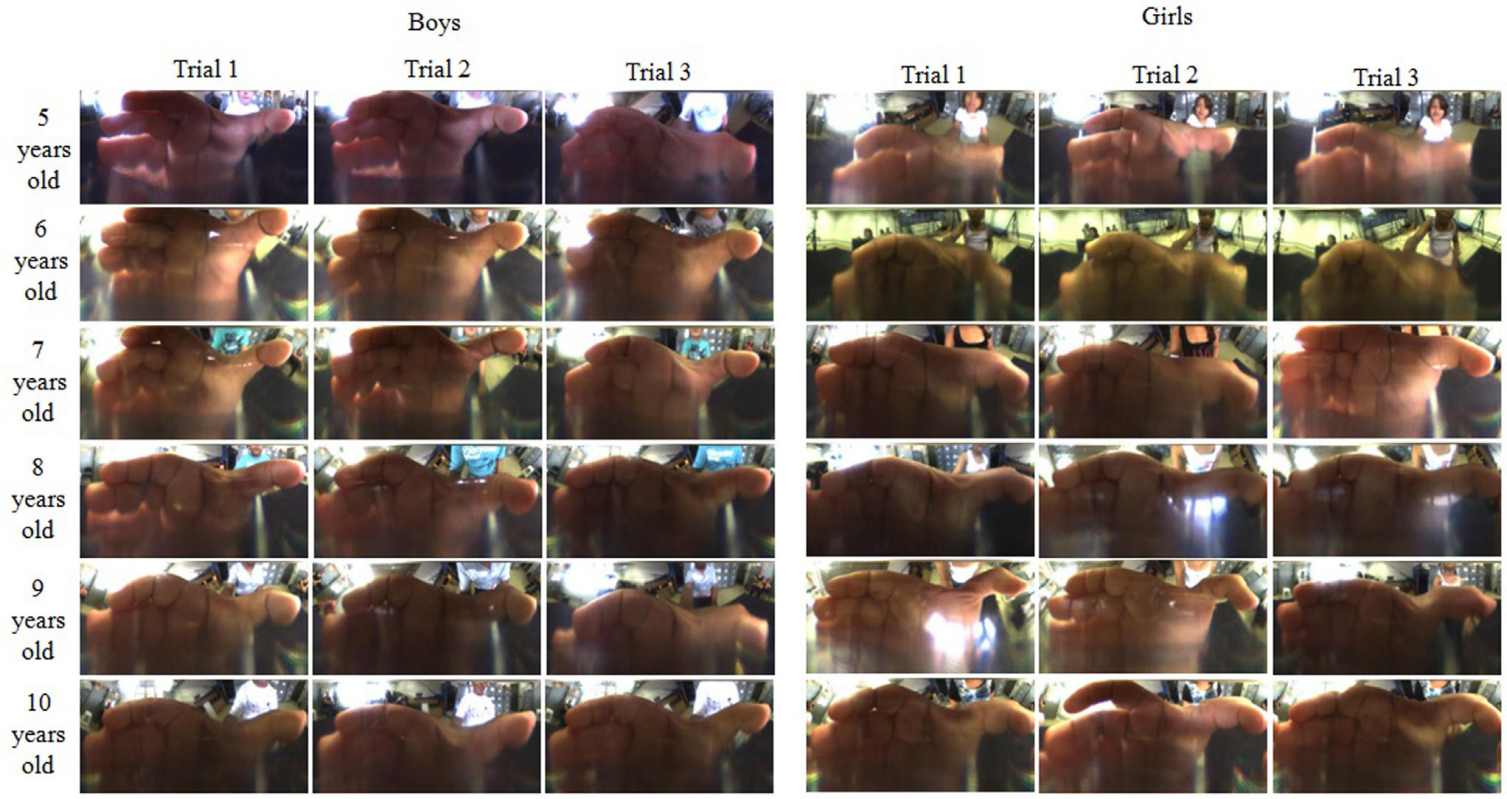
**Hand posture images organized by gender, boys on the left and girls on the right, by trial, 3 for each child, and by age, from 5 up to 10 years old in each line**.

With the access to the hand posture during the trial, it is possible to evaluate its evolution with time, to extract postures in key instants, as the A, B, C, D, and E (shown in Figure 6B) limiting the phases as described in Section “Kinematic and Kinetic Analysis,” and qualitatively evaluate the keys postures among experiment volunteers correlating them to gender, age, and hand dominance. As an example, 36 hand images of hand postures at instant B were selected from 12 children in order to conduct a qualitative evaluation. The images are presented in Figure 8 and are organized by gender, boy on the left and girls on the right, by trial, three for each child, and by age, from 5 to 10 years old in each line.

The images were qualitatively evaluated. For all the postures, the fingers are in a similar position; however, it is possible to notice change of the thumb opposition the fingers. For 5-year-old children, the thumb is opposed to the middle finger, and with the increase of age the thumb becomes opposed to the index finger. Comparing the postures age by age, one can notice the modulation of the thumb opposition to the middle to the index finger. Minor differences could be noticed among trials of a child.

## Discussion and Conclusion

The study of upper limbs motor deficit as a consequence of neurological problems requires the investigation of physical quantities present in specific manipulation functionalities. These measures, being kinematic and kinetic of movements and grasp quality, collaborate with a quantitative assessment of motor deficit, which may lead to the improvement of rehabilitation strategies as well as the establishment of new assessment technologies for rehabilitation.

Usually these physical quantities are obtained by specific motion acquisition systems, for example, camera-based systems with passive or active markers placed over the body to capture movements, dynamometers for the acquisition of grip strength, accelerometers to estimate energy expenditure, among many others already well-established systems. These devices require installation in rooms with special preparation according to the device technology, such as the case of camera-based systems. In these cases, the subjects must move to the location of the device installation. These activities are more difficult for subjects with severe motor impairment. In other cases, where two or more devices are used, the measures are acquired in different repetitions of a task because of the difficulty to integrate and synchronize all the data acquired. In most cases, it is difficult to evaluate a spatio-temporal correlation among data or even data synergies. In addition, similar devices were previously presented by researches, Murgia et al. (2010) presented a device instrumented with an IMU and Tedim Cruz et al. (2014) presented a device equipped with an IMU and an electromyography system. None of them were capable to assess the hand posture.

To evaluate the measurement capacity of the proposed device, experiments were conducted with 47 healthy children of both gender and different ages. Each experimental section had a total duration of 10 min in average, including standard procedures (general guidelines, acceptance term signature, biometric measurements) and execution of the exercise. A total of 23 physical quantities were measured in each trial (22 from the IMU including time and one from the camera). The large number of data acquired in a single trial and the large number of subjects in the pilot experiment presented in this work highlight the potential use of the device in experimental studies and rehabilitation assessment. In addition, the acquisition synchronism of the various measures indicates the capacity for functional analyzes involving spatial and temporal relations and manipulation synergies. In this sense, data from experiments with 47 volunteers children were analyzed according to the existing metrics in the literature as straightness index, energy expenditure, and movement units.

In order to bring the experimental environment to the population of interest in schools and rehabilitations centers in the case of our application, we developed an instrumented portable object. Its design was made as a replica of a daily unimanual object. The object is instrumented with an IMU for kinematic and interaction data acquisition, and, as an innovation in the area, an image acquisition system (consisting of a micro digital camera, a hyperbolic mirror and a cylindrical lens) for measuring characteristics of a hand grasp posture. Data from IMU and camera are synchronously acquired with 87 and 100/60 Hz, respectively. This new assessment system allows us to increase the number of participants in experiments conducted in schools and rehabilitation clinics as well.

In the analysis of kinematic data, it was possible to identify patterns in the spatio-temporal domain as the three different phases of movement: accommodation, transport, and return. The identification of the first phase was performed thanks to the joint analysis of data from accelerometers and from the angular displacement in the sagittal plane. Accelerations analysis identifies the interactions with the object, and in this way, it was possible to determine the instant of contact with the object. Such a moment could not be identified only from data movement, as those obtained by a camera-based systems as quoted before. As a highlight, the accommodation time is suggested as a new opportunity to measure performance and dexterity of upper limbs.

With the orientation in the sagittal plane data and its derivative in respect with time, the rotation speed, it was possible to calculate metrics similar to those classically used in studies in the field: motion units and straightness index. They were calculated using the same equation found in literature references, however, with indirect measures. For calculation of the motion units the rotation speed was used. We admit the hypothesis that these calculations may be strongly related to the classical metrics, or even considered as new metric related to the technology used. New experimental studies are needed to confirm this hypothesis.

Concerning the hand posture analysis, when subjects studied are adults, commercial datagloves are commonly used, however, when the subjects are children the main challenge is to assess hand information since glove sizes available are not suitable to them, among others disadvantage as portability and need of calibration which is time demanding. As an alternative, we presented in this paper a novel device capable of acquiring children hand postures during a specific manipulation task. Such a device is proposed as an additional tool to study manipulation in typical and atypical children.

A preliminary qualitative analysis was performed for 12 children with the aim to present the instrumented object potentiality to assess hand posture. In this evaluation, it was possible to analyze the posture presented by both genders of different ages (from 5 to 10 years). Results suggest the thumb opposition to the fingers is the most important change when postures of children at different ages are compared. Generally, for young children (from 5 to 7 years old) the thumb is most opposed to the middle finger, for children from 8 to 10 years old, the thumb is most opposed to the index finger. No difference between genders was observed. It is hypothesized that the thumb opposition is related to age not to hand size since girls, in general, at the same age girls have smaller hands than boys. Nevertheless, this is a pilot study to evaluate the device, thus statistical analysis have to be conducted to confirm such hypothesis. In addition, it is highlighted that the evaluation was visually performed. With the objective to achieve more conclusive results, a larger number of subjects have to evaluated. Taken into account the great number of images to be analyzed, it is suggested to use an automatic hand posture evaluation as the one presented by Pedro et al. (2011).

Despite the advantages of the proposed device, there are drawbacks. The hyperbolic mirror concentrates visual information close to its center. The digital camera captures the image reflected by the mirror with constant resolution, i.e., with the same size and distribution of the pixels. In this way, information is lost. As a result, it can be observed the low resolution obtained at the bottom of the panoramic images. In some images, the ring and little fingers are not identifiable. As alternative, we suggest the use of higher resolution camera with automatic focus adjustment. Another drawback that may be cited is the inaccuracy of hand posture information during movement of reach-to-grasp, since the hand is not in contact with the glass is not possible to perform quantitative metrics.

Finally, we conclude that the new device presented in this paper can perform measures which such proposed analysis is able to identify and evaluate functional characteristics of upper limbs in typical and atypical children. Results obtained from the pilot experiment indicate this highlighted characteristic. In addition, the device facilitates and reduces demanded time of the experiments increasing the number of subjects and allowing a better statistical analyzes. It is also intended to use the device as a tool in rehabilitation interventions as well as for rehabilitation progress evaluation. It is worth mentioning as device limitations, when compared to conventional camera-based motion acquisition system, it is not able to acquire data during the reach-to-grasp phase. As main future developments, we quote the reduction of size and weight to allow the usage of the device by children younger than 5 years and to potentially conduct early diagnoses of disabilities, and the addition of a pressure sensor and a deformable plastic cylinder substituting the cylinder glass in order to indirect measure the grasp force, correlating the pressure with the grasping area obtained from the panoramic image.

## Author Contributions

All the authors hardly contributed with the writing and review of the manuscript. AR contributed with the experiment with children subjects, data analysis, and statistical analysis. ET author contributes with the ethical approval for the experiments and with the conduction of experiments. LP contributed with kinematic analysis. VA contributed on the vision system method, data analyses, and results. LS contributed with the conception of the device. GC contributed with the mechatronic design of the device presented in the paper.

## Conflict of Interest Statement

The authors declare that the research was conducted in the absence of any commercial or financial relationships that could be construed as a potential conflict of interest.
